# Association between Emotional Symptoms and Job Demands in an Asian Electronics Factory

**DOI:** 10.3390/ijerph14091085

**Published:** 2017-09-19

**Authors:** Wei-Lieh Huang, Yue Leon Guo, Pau-Chung Chen, Jui Wang, Po-Ching Chu

**Affiliations:** 1 Department of Psychiatry, National Taiwan University Hospital Yun-Lin Branch, Yunlin County 640, Taiwan; weiliehhuang@gmail.com; 2 National Institute of Environmental Health Sciences, National Health Research Institutes, Miaoli County 350, Taiwan; leonguo@ntu.edu.tw; 3 Department of Environmental and Occupational Medicine, National Taiwan University and National Taiwan University Hospital, Taipei 100, Taiwan; pchen@ntu.edu.tw; 4 Institute of Occupational Medicine and Industrial Hygiene, College of Public Health, National Taiwan University, Taipei 100, Taiwan; 5 Institute of Epidemiology and Preventive Medicine, College of Public Health, National Taiwan University, Taipei 100, Taiwan; d03849011@ntu.edu.tw; 6 Department of Environmental and Occupational Medicine, National Taiwan University Hospital Hsin-Chu Branch, Hsinchu 300, Taiwan

**Keywords:** job demands, emotional symptoms, electronics industry, clean room, gender

## Abstract

Various work-related issues including mental health have been described for the electronic industry. Although East Asian countries play important roles in the electronics industry, the association between job demands and emotional symptoms has been rarely examined. The present study recruited 603 workers from either office or clean room environments in an electronics factory in Taiwan. Their personal factors, work-related factors, and emotional symptoms were assessed by a self-administered questionnaire. The symptoms of depression and hostility were reported in 24.88% and 24.38% of the subjects, respectively, while 14.93% reported both. A multivariate analysis showed that, overall, women workers were more likely to have emotional symptoms than male workers (odds ration (OR) = 1.50, 95% CI = 1.02–2.18). Among clean room workers, working under high pressure (OR = 1.84, 95% CI = 1.05–3.21), conflicting demands (OR = 2.15, 95% CI = 1.30–3.57), and social isolation at work (OR = 2.99, 95% CI = 1.23–7.30) were associated with emotional symptoms. The findings suggest that in the Asian electronics industry, for women, working under high pressure, conflicting demands, and social isolation at work are risk factors for emotional symptoms, especially for clean room workers. Further large-scale, longitudinal studies are necessary to confirm and prevent the mental health problems in this fast-evolving, highly competitive industry.

## 1. Introduction

The electronics industry was estimated to employ 18 million workers worldwide in 2010 [1], and East Asian countries including Japan, South Korea, and Taiwan contributed to over 50% of the global export values. Contrary to the high-tech, clean image of the industry, the work environment may contain chemical hazards (i.e., irritants, allergens, metals, fiberglass), physical hazards (i.e., low humidity, noise, nonionizing radiation), and ergonomic hazards (i.e., repetition, lighting, standing posture) [2,3,4]. Chronic exposure to these hazards has been linked to possible adverse outcomes such as systematic toxicity, issues in reproductive health, and increased risk of cancer [3]. Electronic manufacturing often takes place in clean rooms, where employees are required to be fully covered in protective suites [5]. When the workers remain fully suited during the entire work shift, these head-to-toe garments can cause inconvenience and discomfort. In addition, the industry is known for rapid technological innovation, global competition, operating on shift work, high job demands, and performance-based pay systems [3,6]. All of the aforementioned factors may negatively affect the mental health of the employees. However, work-related mental problems are often overlooked due to the high confidentiality requirement and the great economic importance of the industry.

Mental disorders are associated with occupational dysfunction, long-term sickness absence, loss of productivity, poverty, social isolation, and economic burden (i.e., the payment of disability benefits) [7]. Workers with mental disorders are likely to experience a range of emotional symptoms that can impair their performance at work and increase the risk of accidents [8]. Chronic work-related strains are known risk factors of mental disorders. A longitudinal, prospective cohort study found that job strain arising from high job demands was associated with mental disorders among London based civil servants [9]. Analysis of the national workers’ compensation database in Australia showed that the most common reasons for work-related mental health claims (41%) were work responsibilities, job demands, interpersonal conflicts in the workplace, etc. [10]. Bromet et al. [11] found that high job demands combined with lack of social support at work increase the risk of depression and anxiety. In addition to work-related factors, the risk factors for mental disorders or emotional symptoms also include non-work-related factors such as gender, lifestyle, etc. Women have a higher prevalence of depression than men, and this gender difference may be explained by genetic, neurohormonal, psychobiological, and social factors [12,13]. Alcohol drinking and cigarette smoking are associated with major depression and other emotional symptoms [14]. In addition, regular exercise is a protective factor to depression or anxiety [15]. It is crucial to identify early signs of mental distress to prevent the severe consequences of mental illnesses at the levels of the individual and the society.

East Asian countries such as Taiwan and Japan are reported to have a lower prevalence rate of depressive and anxiety disorders than do western societies [16]. The lifetime prevalence of major depression in Taiwan (1.14%) is lower than that in the United States (5.15%) [17], and the 12-month prevalence rate of major depression is 0.8% in Taiwan [18]. However, “cultural stoicism” in Taiwanese adults may result in high diagnosis thresholds and low help-seeking behavior s compared with that of the adult population in western countries [19]. Individuals in Taiwan with mental problems sometimes complain only of physical symptoms (i.e., pain) [20]. Thus, proactive surveillance is likely necessary to identify the individuals who are at risk. However, few existing reports studied the prevalence of mental problems or emotional symptoms in the electronics industry in Taiwan or other Asian countries. Studies found that high job demands were associated with unhealthy mental states (such as depressive disorders) for this industry [21,22,23], but how clean rooms as a work environment affects the workers’ mental health is still not well understood. A Chinese study found that male clean-room workers had higher work stress than their women counterparts, and clean-room workers had higher work stress than non-clean-room workers [5], but more studies are necessary to validate the findings. Taken together, it is critically important to understand the emotional symptoms among electronics workers in Taiwan, especially considering unique characteristics of the industry and culture. As a proof-of-concept study, the aim of this study is to analyze potential risk factors to determine whether they have an independent influence on the emotional symptoms of Taiwanese workers in a representative electronics factory.

## 2. Study Population and Methods

### 2.1. Study Population

**Figure 1:**
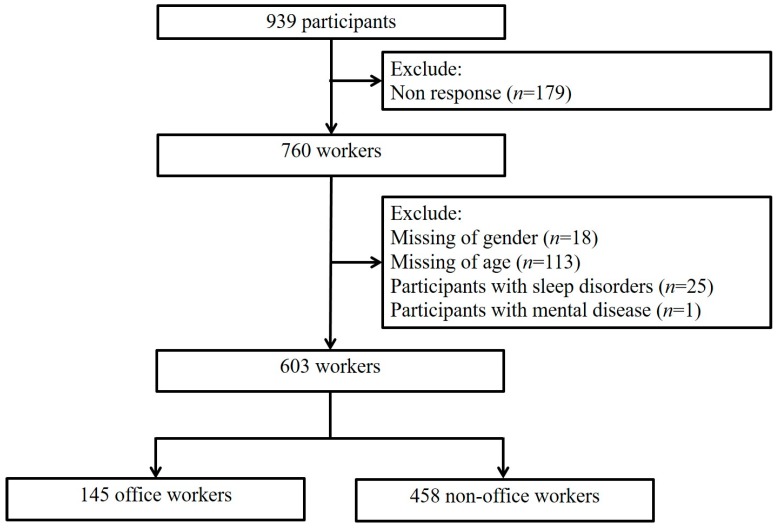
Flow chart of recruiting study population.

A questionnaire survey with a cross-sectional design was conducted. The study sample was drawn from an electronics enterprise in 2011. All available participants at the enterprise were invited to participate in the assessment. Formal written instructions for the survey were posted in the workplace, and a research staff member presented a verbal briefing based on a written script to the participants prior to the distribution of the questionnaires. The participants were given the opportunity to decline participation or to withdraw at any time. Privacy was guaranteed during the survey, and the survey was anonymous. A total of 939 participants were surveyed (Figure 1), and the response rate was 80.90%. Participants who had missing values for gender (*n* = 18) or age (*n* = 113) were excluded from the analyses. Because workers with pre-existing mental disorders may over-report unfavorable work characteristics [24], workers with previous sleep disorders (*n* = 25) or mental disease (*n* = 1) were also excluded. A final of 603 participants remained in the study population, including clean-room workers (*n* = 458) and office workers (*n* = 145). Ethical approval for the study was granted by the National Taiwan University Hospital Ethics Committee (201301059RINC).

### 2.2. Assessment of Personal and Work-Related Factors

The self-administered questionnaire items contained two parts: (1) personal factors, including basic demographic data (i.e., age, gender) and lifestyle habits (i.e., cigarette smoking, alcohol drinking, regular exercise); and (2) work-related factors, including the work environment (clean room vs. office), the duration of employment, temporal work patterns (i.e., shift work, night work), and job demands. The assessment of job demands was based on “Hazard: stress at work” from the “Risk Assessment Essentials of the European Agency for Safety and Health at Work”. The face validity was applied to estimate the understanding and acceptance of the questionnaire by the target population [25]. The face validity of the items related to job demands was assessed by a pilot study with ten men and ten women participants from the electronics industry. The items and scales were modified based on the participants’ comments. The final job demand questionnaire consisted of nine items on a two-point categorical scale (“Yes, rather often”, and “no, seldom”). The items were: (1) Do employees usually or occasionally work under high pressure (fast pace of work, tight deadlines)? (2) Do employees usually or occasionally work long hours? (3) Is the workload usually or occasionally very high? (4) Is there a balance between physical and mental job demands and workers’ abilities? (5) Is the job monotonous? (6) Are there physical risks (noise, temperature, chemicals, etc.)? (7) Are workers clear about what their duties are? (8) Do workers have conflicting demands? (9) Are workers socially isolated when doing their job?

### 2.3. Outcome Measures

Because the industry is highly competitive and requires a high level of information security, it was impossible to administer detailed mental health questionnaires, such as the Beck Depression Inventory [26] or the Center for Epidemiological Studies Depression Scale [27]. Thus, the 5-item Brief Symptom Rating Scale (BSRS-5) was used as a surrogate. BSRS-5 is commonly used for screening psychological disorders in Taiwan, and has been shown to have good validity and reliability in work environments [28]. It contains five items to measure psychological symptoms including anxiety (feeling tense or high-strung), depression (feeling depressed or in a low mood), hostility (feeling easily annoyed or irritated), inferiority (feeling inferior to others), and sleep disturbance (having trouble falling asleep in the past week). Because depression in the electronics industry has been reported in previous studies [21,23], depressive symptoms were regarded as a major outcome of interest in the present study. Another study showed that hostility was closely associated with depression in the working population [29]. Therefore, symptoms of hostility were also regarded as a major outcome.

### 2.4. Data Analysis

The distribution of emotional symptoms according to basic characteristics were analyzed by chi-square tests for categorical variables (gender, work environment, and lifestyle) and by 2-sample independent t-tests for continuous variables (age). The distribution differences of emotional symptoms according to the items of job demands were also analyzed by chi-square test. Univariate logistic regression analyses were performed to investigate the possible associations between emotional symptoms and work-related factors, non-work-related factors, or items of job demands. Multivariate logistic regression analyses were then performed to adjust for age, gender, and other variables that showed significant association in the univariate analyses. Gender and work environment were selected for stratified analyses, followed by multivariate logistic regression analyses to adjust for variables having significant association in the univariate analyses. Even though work environment was not a significant factor for emotional symptoms in our univariate analysis, it was chosen for further analysis because work-related stress was found to be different between clean-room versus non-clean-room workshops [5]. Odds ratios (OR) and the corresponding 95% confidence intervals (CI) were calculated, and a *p*-level < 0.05 was considered statistically significant. All analyses were performed using SAS software version 9.4 (SAS Institute, Cary, NC, USA).

## 3. Results

### 3.1. Participant Characteristics

The basic characteristics and lifestyle factors of the study population are presented in Table 1. We identified 603 participants, including 145 workers (24.05%) in the office environment and 458 workers (75.95%) in the clean room environment. Among the participants, 207 workers (34.33%) reported emotional symptoms, and 396 workers (65.67%) did not. Of those with emotional symptoms, 150 (24.88%) had depressive symptoms, 147 (24.38%) had symptoms of hostility, and 90 (14.93%) had both types of emotional disturbance.

The mean ages were 30.4 ± 4.9 and 30.3 ± 5.1 years for people with and without emotional symptoms, respectively. There was a higher proportion of women workers with emotional symptoms compared with those without emotional symptoms (61.35% vs. 52.53%, respectively, *p* = 0.038). Between workers with and without emotional symptoms, there was no significant differences in work environment (*p* = 0.518), shift work (*p* = 0.913), or night work (*p* = 0.115). The two groups of workers were also similar in cigarette smoking (*p* = 0.203) and alcohol consumption (*p* = 0.158). There were 26.52% of workers without emotional symptoms who exercised regularly, which was significantly higher than 15.94% among those with emotional symptoms (*p* = 0.003).

### 3.2. Correlates of Emotional Symptoms

Table 2 summarized the job demands reported by the workers with and without emotional symptoms. Compared with workers without emotional symptoms, those with emotional symptoms had significantly higher rates of working under high pressure (78.74%), working long hours (77.78%), high workloads (69.08%), imbalances between physical/mental job demands and the worker’s abilities (42.51%), conflicting demands (38.65%), and social isolation at work (11.59%; all *p*-values < 0.001).

Logistic regression analysis was performed to estimate the crude and multivariable-adjusted odds ratios and to identify factors associated with emotional symptoms (Table 3). The presence of emotional symptoms was positively correlated with being a woman (OR = 1.44, 95% CI = 1.02–2.02) and negatively correlated with regular exercise (OR = 0.53, 95% CI = 0.34–0.81). In terms of job demands, working under high pressure, working long hours, high workloads, imbalances between physical/mental job demands and the worker’s abilities, conflicting demands, and social isolation at work were significantly correlated with emotional symptoms. Age, gender, and factors that were significantly associated with emotional symptoms were included in the multiple logistic regression model. Being a woman (OR = 1.50, 95% CI = 1.02–2.18) and regular exercise (OR = 0.59, 95% CI = 0.37–0.94) were still associated with having emotional symptoms, whereas the job demand factors that remained significant included working under high pressure (OR = 1.88, 95% CI = 1.17–3.02), conflicting demands (OR = 1.77, 95% CI = 1.15–2.73), and social isolation at work (OR = 2.44, 95% CI = 1.14–5.22). Interactions were further examined for two aspects: (1) gender and job demands and (2) work environment and job demands. There were no statistically significant interactions between gender and job demand factors that were significant in simple logistic regression, including working under high pressure, working long hours, high workload, imbalance between physical/mental job demands and workers’ abilities, conflicting demands, and social isolation at work. In addition, there were also no interactions between work environment and these job demands factors.

### 3.3. Gender Effect on Correlates of Emotional Symptoms

To identify possible gender-specific factors, the workers were stratified by gender and the results are shown in Table 4. For men, after adjustment for other variables, only alcohol consumption (OR = 2.43, 95% CI = 1.04–5.67) and social isolation at work (OR = 3.07, 95% CI = 1.12–8.42) were significantly associated with emotional symptoms. For women workers, working under high pressure (OR = 2.31, 95% CI = 1.12–4.76) and conflicting demands (OR = 2.10, 95% CI = 1.15–3.84) were significant factors of having emotional symptoms.

### 3.4. Effect of Work Environment on Correlates of Emotional Symptoms

We further conducted stratified analysis by work environment. Compared with office workers, clean room workers were younger, more of them were men, had shift work or night work, and had the habit of smoking (Table 5). The factors influencing emotional symptoms for office vs. clean room workers are shown in Table 6. For office workers, shift work, working under high pressure, working long hours, high workloads, and imbalances between physical/mental job demands and the worker’s abilities had significant associations with emotional symptoms, but none of these factors were significant in the multivariable regression. Among the clean room workers, regular exercise (OR = 0.51, 95% CI = 0.30–0.88), working under high pressure (OR = 1.84, 95% CI = 1.05–3.21), conflicting demands (OR = 2.15, 95% CI = 1.30–3.57), and social isolation at work (OR = 2.99, 95% CI = 1.23–7.30) were significantly associated with emotional symptoms in the multiple logistic regression model.

## 4. Discussion

In the present study, we explored the mental conditions in two different types of work environments in an electronics manufacturing factory: the office environment and the clean room environment. For office workers, working under high pressure, working long hours, a high workload, and an imbalance between physical and mental job demands and the worker’s abilities were strongly associated with emotional symptoms. For clean room workers, regular exercise was a protective factor, which is similar to the findings of the following two studies: Sugisawa et al. [30] who found that both work load and health practices (i.e., exercise) had a significant independent influence on mental health after adjusting for age and marital status, and Nomura et al. [15] who showed that healthy behaviors (regular exercise, a calorie-focused diet, or both) were negatively associated with higher job demands (OR = 0.69, 95% CI = 0.53–0.89) after adjusting for age, lifestyle, and health beliefs.

We noticed that for clean room workers, working under high pressure, facing conflicting demands, and social isolation at work were significantly associated with emotional symptoms after adjustment for age, gender, and other job demands, whereas no such correlations were disclosed in office workers. In the present study, the characteristics of clean room workers were young age, high proportion of men workers, high proportion of smoking, and many workers with shift work or night work, compared with that of office workers (Table 5). These discrepancies may account for the different association with emotional symptoms between office workers and clean room workers. The finding regarding working under high pressure was consistent with a previous report which found that the risk of stress in clean workshops was higher than that in ordinary workshops in an electronics company [5]. The high percentage of conflicting demands in the clean room environment may be associated with the fast-changing nature of the work, which often requires the workers to learn new knowledge or skills to adapt to new manufacturing processes in this constantly evolving industry. Workers in the clean room environment had to wear clean caps, protective suits, masks, gloves, and shoes [5]. These clothing and protective gears may increase the difficulty of interpersonal communication, which may explain the social isolation of these workers.

We explored the gender differences in emotional symptoms and the associated factors and found that the women workers suffered more emotional symptoms than their male counterparts. The emotional symptoms in women workers were significantly associated with working under high pressure and conflicting demands after adjustment for age, lifestyle, and other job demands. This finding was similar to that of a previous study that showed women workers reporting more conflicting demands than men workers [31].

There are several potential limitations to the present study, mostly due to the characteristics of the electronics industry. First, the participants in this study were recruited from one electronics enterprise. Although the enterprise was selected based on its representative characteristics of the general electronics industry, it is nonetheless a potential bias of this study. Second, because the requirement of confidentiality is high in the electronics industry, long-term follow-up of workers with mental problems was not possible, leading to the cross-sectional design of the present study. Thus, we could not monitor the progression of emotional symptoms or deduce the direction of cause for the emotional symptoms assessed. Third, although we adjusted for potential confounders including age, gender, lifestyles, etc., information regarding job control and support, personality, coping, long-term emotional symptoms, financial difficulties (i.e., low salary), living alone, negative life events [24], as well as mediating appraisals (i.e., stress and job satisfaction) were not examined in this study. Fourth, because of the time constraints due to a tight production schedule, the instruments for diagnosing mental disorders and assessing job demands had to be simplified. Although using emotional symptoms as outcomes may reveal early warning signs of later disease development, the workers with symptoms of depression may or may not have depressive disorder. It is worth noting that, in the present study, 25% of the workers reported depression symptoms, which was close to the previously reported depression rates for clean workshop (24.8%) and ordinary workshop (23.8%) workers of a Chinese electronics factory assessed by the Center for Epidemiologic Studies Depression Scale (CES-D) [5]. This result suggests that our survey likely represents a valid estimation of the real situation, and may be used as evidence to persuade industrial owners and the government to comply and support a large-scale, long-term study in the future.

## 5. Conclusions

To conclude, for women, working under high pressure, conflicting demands, and social isolation at work may be risk factors for emotional symptoms in this electronics industry. The emotional symptoms of women workers were associated with conflicting demands. Workers who experienced conflicting demands and social isolation at work in clean room showed much higher levels of emotional symptoms. Future large-scale studies with longitudinal follow-up are needed to further elucidate the impacts of gender, job demands, and work environment on the emotional symptoms and mental disorders of workers in the electronics industry.

## Figures and Tables

**Table 1:** Basic characteristics of study population and distribution of emotional symptoms.

Variables	Emotional Symptoms		
	Without	With	*p*-Value ^1^
	*n* = 396	*n* = 207	
Age (years)	30.3 ± 5.1	30.4 ± 4.9	0.940
Gender			0.038
Women	208 (52.53%)	127 (61.35%)	
Men	188 (47.47%)	80 (38.65%)	
Work environment			0.518
Office	92 (23.23%)	53 (25.60%)	
Clean room	304 (76.77%)	154 (74.40%)	
Shift work	128 (32.32%)	66 (31.88%)	0.913
Night work	152 (38.38%)	66 (31.88%)	0.115
Lifestyle			
Smoking	125 (31.57%)	76 (36.71%)	0.203
Alcohol	24 (6.06%)	19 (9.18%)	0.158
Regular exercise	105 (26.52%)	33 (15.94%)	0.003

^1^ Student’s *t*-test and Chi-squared test.

**Table 2:** Distribution of job demands for emotional symptoms according to job demands.

Job Demands	Emotional Symptoms		
	Without	With	*p*-Value ^1^
	*n* = 396	*n* = 207	
Working under high pressure	212 (53.54%)	163 (78.74%)	<0.001
Working long hours	227 (57.32%)	161 (77.78%)	<0.001
High workload	183 (46.21%)	143 (69.08%)	<0.001
Imbalance between physical/mental job demands and workers’ abilities	113 (28.54%)	88 (42.51%)	<0.001
Monotonous job	114 (28.79%)	73 (35.27%)	0.103
Presence of physical risks	195 (49.24%)	111 (53.62%)	0.307
Unclear understanding about job duties	9 (2.27%)	3 (1.45%)	0.492
Conflicting demands	78 (19.95%)	80 (38.65%)	<0.001
Social isolation at work	14 (3.54%)	24 (11.59%)	<0.001

^1^ Chi-squared test.

**Table 3:** Univariate and multivariate logistic regression analysis of factors influencing emotional symptoms.

Variables	Emotional Symptoms			
	Crude		Multivariable ^4^	
	OR	95% CI	OR	95% CI
Age (years)	1.00	0.97–1.04	0.98	0.95–1.02
Women	1.44	1.02–2.02 ^1^	1.50	1.02–2.18 ^1^
Work environment	1.14	0.77–1.68	-	-
Shift work	0.98	0.68–1.41	-	-
Night work	0.75	0.53–1.07	-	-
Smoking	1.26	0.88–1.79	-	-
Alcohol	1.57	0.84–2.93	-	-
Regular exercise	0.53	0.34–0.81 ^2^	0.59	0.37–0.94 ^1^
Working under high pressure	3.22	2.18–4.74 ^3^	1.88	1.17–3.02 ^2^
Working long hours	2.61	1.78–3.82 ^3^	1.48	0.91–2.42
High workload	2.60	1.82–3.71 ^3^	1.34	0.84–2.14
Imbalance between physical/mental job demands and workers’ abilities	1.85	1.30–2.63 ^2^	1.15	0.78–1.70
Monotonous job	1.35	0.94–1.93	-	-
Presence of physical risks	1.19	0.85–1.67	-	-
Unclear understanding about job duties	0.63	0.17–2.36	-	-
Conflicting demands	2.53	1.74–3.67 ^3^	1.77	1.15–2.73 ^2^
Social isolation at work	3.58	1.81–7.08 ^2^	2.44	1.14–5.22 ^1^

Note: OR = Odds Ratio; CI = Confidence interval.^1^: *p* < 0.05; ^2^: *p* < 0.01; ^3^: *p* < 0.0001. ^4^: Multivariable model included: age, gender and independence of risk factors in the univariate analysis.

**Table 4:** Univariate and multivariate logistic regression analysis of factors influencing emotional symptoms, stratified by gender.

Variables	Men (*n* = 268)				Women (*n* = 335)			
	Crude		Multivariable ^3^		Crude		Multivariable ^3^	
	OR	95% CI	OR	95% CI	OR	95% CI	OR	95% CI
Age(years)	1.03	0.98–1.09	1.02	0.96–1.08	0.98	0.94–1.02	1.02	0.96–1.08
Work environment	0.93	0.43–2.04	-	-	1.08	0.67–1.72	-	-
Shift work	0.56	0.31–1.01	-	-	1.47	0.92–2.36	-	-
Night work	0.73	0.43–1.24	-	-	0.83	0.51–1.35	-	-
Smoking	1.76	1.04–3.00 ^1^	1.31	0.73–2.36	1.21	0.71–2.06	-	-
Alcohol	2.87	1.31–6.27 ^2^	2.43	1.04–5.67 ^1^	0.64	0.20–2.10	-	-
Regular exercise	0.52	0.29–0.96 ^1^	0.55	0.28–1.05	0.60	0.32–1.14	-	-
Working under high pressure	3.48	1.89–6.38 ^2^	2.09	0.98–4.46	3.00	1.81–4.97 ^2^	2.31	1.12–4.76 ^1^
Working long hours	2.81	1.53–5.16 ^2^	1.73	0.77–3.92	2.48	1.51–4.08 ^2^	1.72	0.79–3.76
High workload	2.33	1.35–4.02 ^2^	0.91	0.43–1.94	2.78	1.74–4.45 ^2^	0.97	0.47–1.99
Imbalance between physical/mental job demands and workers’ abilities	1.65	0.96–2.86	-	-	1.99	1.26–3.16 ^2^	0.79	0.53–1.80
Monotonous job	1.06	0.60–1.89	-	-	1.55	0.97–2.47	-	-
Presence of physical risks	1.41	0.82–2.40	-	-	1.14	0.73–1.78	-	-
Unclear understanding about job duties	1.18	0.21–6.57	-	-	0.32	0.04–2.79	-	-
Conflicting demands	2.71	1.57–4.70 ^2^	1.60	0.84–3.06	2.72	1.60–4.61 ^2^	2.10	1.15–3.84 ^1^
Socially isolation at work	5.19	2.10–12.82 ^2^	3.07	1.12–8.42 ^1^	2.57	0.89–7.39	-	-

Note: OR = Odds Ratio; CI = Confidence Interval. ^1^: *p* < 0.05; ^2^: *p* < 0.01. ^3^: Multivariable model included: age and independence of risk factors in the univariate analysis.

**Table 5:** Comparison of demographic and employment characteristics between office workers and clean room workers.

Variables	Office	Clean Room	*p*-Value ^1^
	*n* = 145	*n* = 458	
Age (years)	32.5 ± 4.2	29.6 ± 5.0	<0.001
Gender			<0.001
Women	110 (75.86%)	225 (49.13%)	
Men	35 (24.14%)	233 (50.87%)	
Shift work	28 (19.31%)	166 (36.24%)	<0.001
Night work	11 (7.59%)	207 (45.20%)	<0.001
Lifestyle			
Smoking	25 (17.24%)	176 (38.43%)	<0.001
Alcohol	8 (5.52%)	35 (7.64%)	0.386
Regular exercise	31 (21.38%)	107 (23.36%)	0.620

^1^ Student’s *t*-test and Chi-squared test.

**Table 6:** Univariate and multivariate logistic regression analysis of factors influencing emotional symptoms, stratified by work environment.

Variables	Office (*n* = 145)				Clean Room (*n* = 458)			
	Crude		Multivariable ^3^		Crude		Multivariable ^3^	
	OR	95% CI	OR	95% CI	OR	95% CI	OR	95% CI
Age (years)	0.97	0.89–1.05	0.93	0.84–1.02	1.01	0.97–1.05	0.99	0.95–1.03
Women	1.60	0.70–3.67	1.13	0.46–2.81	1.39	0.94–2.05	1.49	0.97–2.30
Shift work	2.40	1.04–5.54 ^1^	1.93	0.77–4.86	0.81	0.54–1.22	-	-
Night work	0.99	0.28–3.56	-	-	0.74	0.50–1.10	-	-
Smoking	1.46	0.61–3.50	-	-	1.27	0.86–1.89	-	-
Alcohol	1.80	0.43–7.50	-	-	1.53	0.76–3.08	-	-
Regular exercise	0.79	0.34–1.83	-	-	0.46	0.28–0.77 ^2^	0.51	0.30–0.88 ^1^
Working under high pressure	3.82	1.75–8.33 ^2^	2.30	0.88–6.02	3.05	1.95–4.76 ^2^	1.84	1.05–3.21 ^1^
Working long hours	3.36	1.59–7.09 ^2^	1.23	0.46–3.33	2.42	1.55–3.80 ^2^	1.49	0.84–2.64
High workload	3.79	1.81–7.92 ^2^	2.18	0.82–5.83	2.31	1.54–3.47 ^2^	1.13	0.66–1.96
Imbalance between physical/mental job demands and workers’ abilities	2.27	1.12–4.60 ^1^	1.52	0.68–3.40	1.73	1.15–2.59 ^2^	1.03	0.65–1.63
Monotonous job	1.34	0.57–3.19	-	-	1.40	0.93–2.09	-	-
Presence of physical risks	0.67	0.31–1.46	-	-	1.48	0.99–2.20	-	-
Unclear understanding about job duties	0.42	0.05–3.89	-	-	0.79	0.15–4.10	-	-
Conflicting demands	1.50	0.73–3.09	-	-	3.04	1.96–4.70 ^2^	2.15	1.30–3.57 ^2^
Socially isolation at work	1.42	0.36–5.54	-	-	4.89	2.17–11.03 ^2^	2.99	1.23–7.30 ^1^

Note: OR = Odds Ratio; CI = Confidence Interval. ^1^: *p* < 0.05; ^2^: *p* < 0.01. ^3^: Multivariable model included: age and independence of risk factors in the univariate analysis.
